# A Matrix Metalloproteinase Mediates Tracheal Development in *Bombyx mori*

**DOI:** 10.3390/ijms22115618

**Published:** 2021-05-25

**Authors:** Yi Wei, Xiao-Lin Zhou, Tai-Hang Liu, Peng Chen, Xia Jiang, Zhan-Qi Dong, Min-Hui Pan, Cheng Lu

**Affiliations:** 1 State Key Laboratory of Silkworm Genome Biology, Southwest University, Chongqing 400716, China; weiyi616@yeah.net (Y.W.); zhouxl@ucas.ac.cn (X.-L.Z.); liuth@cqmu.edu.cn (T.-H.L.); pjchen@swu.edu.cn (P.C.); jiangxia1203@yeah.net (X.J.); zqdong@swu.cn (Z.-Q.D.); 2 Key Laboratory for Sericulture Functional Genomics and Biotechnology of Agricultural Ministry, Southwest University, Chongqing 400716, China

**Keywords:** *Bombyx mori*, trachea, extracellular matrix, matrix metalloproteinases

## Abstract

The trachea of insects is a tubular epithelia tissue that transports oxygen and other gases. It serves as a useful model for the studying of the cellular and molecular events involved in epithelial tube formation. Almost all of the extracellular matrix can be degraded by Matrix metalloproteinases (MMPs), which is closely related to the processes of development and regeneration. The regulation of trachea by MMPs is roughly known in previous studies, but the detailed regulation mechanism and involved gene function are not fully explored. In this article, we found MMP1 expressed highly during tracheal remodeling, and knocked out it makes the tracheal branch number reduced in *Bombyx mori*. In trachea of transgenic BmMMP1-KO silkworm, the space expanding of taenidium and epidermal cells and the structure of apical membrane were abnormal. To explore the underlying mechanism, we detected that DE-cadherin and Integrin β1 were accumulated in trachea of transgenic BmMMP1-KO silkworm by immunohistochemistry. Moreover, 5-Bromo-2′-Deoxyuridine (BrdU) labeling showed that knockout of BmMMP1 in silkworm inhibited tracheal cell proliferation, and BmMMP1 also regulated the proliferation and migration of BmNS cells. All of the results demonstrated that BmMMP1 regulates the development of the tracheal tissue by expanding the space of tracheal cuticles and increases the number of tracheal branches by degrading DE-cadherin and Integrin β1.

## 1. Introduction

Matrix metalloproteinase is a kind of multifunctional zinc ion-dependent endopeptidase [1]. MMPs have captured attention because of their high expression in many human pathologies and tissue remodeling [2,3], which is closely related to their ability in degrading almost all of the extracellular matrix (ECM) [1,4]. The ECM is involved in cell adhesion, cell signaling and the structural maintenance of tissues, as a complex network of proteins and proteoglycans [2,5]. During tissue remodeling, ECM components, cell–cell junctions and cell–ECM junctions can be degraded by MMPs [6,7]. DE-cadherin plays a key role in adheren junctions to recruit other adhesion proteins to cytoskeleton nucleation sites [8]. Integrins are major adhesion proteins in cell–ECM junctions and act to couple the ECM components to the cytoskeleton [9]. In the processes of MMPs regulating the degradation and remodeling of ECM components, cells growth also can be affected, such as cell proliferation and motility [7,10,11,12].

Compared with a large number of MMPs in mammals (more than twenty) [13], there are few numbers of MMPs in insects (less than three in each species), so the study of MMPs in insects is more convenient and accurate than in mammals [6,14,15]. In addition, insects have other advantages of short growth cycle, large number of offspring, clear genetic background and easy access. Therefore, usage of insects as the model for studying the functions of MMPs could help to analyze the roles of MMPs in tissue development more quickly.

Insect organs undergo dynamic morphogenesis during metamorphosis [14,15], which include the renewal of the trachea at each new instar [16,17]. The trachea of insects is the gas exchange site for muscles enabling flight and directed movement. Additionally, the trachea also functions to promote metabolism and tissue development [18,19]. The evolution of the tracheal system helped terrestrial arthropods to expand their range of habitats and increase their morphological diversity [18,20]. The tracheal tubes are lined with a barrier of apical (lumenal) chitin ECM that is continuous with the exoskeletal cuticle [21,22]. Almost every protein component of the ECM can be cleaved by MMPs, which suggesting that MMPs may be involved in tracheal development [2]. For instance, MMP1 is essential for the normal growth of the trachea, which is stretched and broken as the larvae grow of MMP1 mutant in *Drosophila melanogaster* [16]. The regulatory interplay between MMPs and FGF signaling operates broadly in invasive growth and branching morphogenesis [17]. Although some functions of MMPs are known, the mechanism of MMPs in mediating tracheal development in *D. melanogaster* is remain unclear. Therefore, it is necessary to conduct comparative research with other insect species.

*Bombyx mori* has a large tracheal branch network. Morphogenesis takes place in the 25th embryonic stage [23] by ectoderm invagination, and then develops into 10 pairs of tracheal plexus, which are distributed on both sides of the body. While the spiracle of the pair in the junction of the second and third segments of the abdomen wound degenerated in the process of growth, nine pairs of trachea plexus for silkworm are also considered. The trachea from the tracheal plexus branches repeatedly and extends into the tissues and cells to form a complete tracheal network, which enables oxygen to diffuse throughout the body. The silkworm trachea consists of a basal cell surface, epithelium, apical cell surface and taenidia. The combination of the apical cell surface and taenidia is the cuticle, which is a non-cellular structure with a thin layer of chitin, numerous proteins and carbohydrates secreted by the epithelium, as a specialized ECM, and shed during molting, and MMP mediates such ECM remodeling events that are required for this organ system to grow.

In this article, we have found that tracheal branch number of BmMMP1-KO mutants was reduced and the mutants almost all died after the third day of the fifth larval instar. It illustrated that a matrix metalloproteinase, MMP1, is required for normal tracheal growth in the silkworm. Which is different from the phenotypes of MMPs knockout in *D. melanogaster*. It is suggested that it can be used as a new model for studying the regulation of MMPs in the development of the trachea. Therefore, studying the mechanism of BmMMP1-KO phenotypic differences in the silkworm will explain the function of MMPs and provide a reference for exploring the developmental mechanism of trachea in animals.

## 2. Results

### 2.1. BmMMP1 Was Highly Expressed during the Critical Remodeling Periods of the Trachea

**Figure 1:**
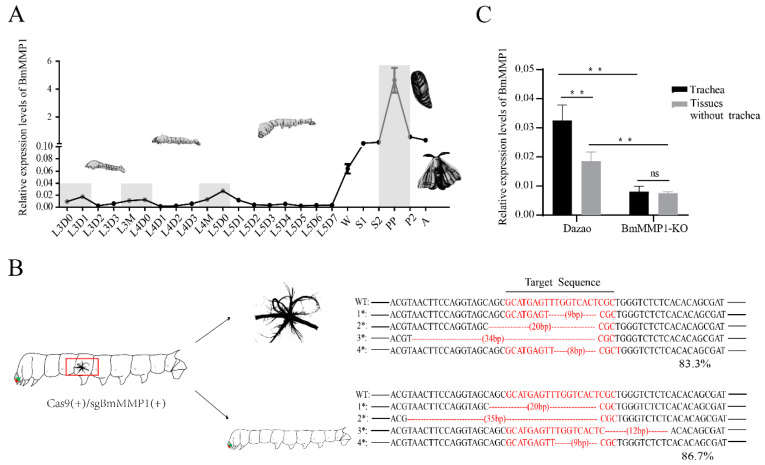
Analysis of expression characteristics of BmMMP1 in silkworm trachea. (**A**) The expressions of BmMMP1 mRNA levels during trachea development in *B. mori*. L3–L5, third to fifth larval instars; W, wandering stage; S, Spinning stage; PP, prepupal stage; P, pupal stage; A, adult stage. The gray rectangles were used to mark the BmMMP1 highly expressed periods. The morphology of silkworm at each stage appears in the corresponding developmental stage. (**B**) DNA sequencing analysis of the CRISPR/Cas9 editing BmMMP1 in trachea and tissues without trachea. 83.3% represented the knockout efficiency of BmMMP1 in the trachea and 86.7% represented that in tissues without trachea. The target sequence as the mark shows. (**C**) The expressions of BmMMP1 mRNA levels in the trachea and tissues without trachea of WT and mutants. The samples in results B&C are silkworms in L5D1. (ns, *p* ≥ 0.05; * *, *p* < 0.01).

The mRNA expression level of BmMMP1 was detected, and the result showed the expression was high in the critical remodeling period of the trachea, which is marked by gray rectangles (Figure 1A). The first three periods are near interlarval molts, and the last is the pre-pupal stage. On the contrary, the two other members of BmMMPs do not show such specificity in tracheal development (Figure S1A,B). In a previous study, we established BmMMP1-KO mutants through CRISPR/Cas9-mediated gene editing (unpublished data), and the principle of the establishment of the strain was described, and the results were explained in supplementary data (Figure S1C). Remarkably, the mutants had no obvious tissue abnormalities except for trachea, and died largely at the fifth instar larvae. The Cas9(+)/sgBmMMP1(+) transgenic hybrid line is BmMMP1-KO mentioned in this study.

Firstly, to assure that MMP1 was edited by CRISPR/Cas9-mediated gene editing and rule out the difference of knockout efficiency between trachea and other tissues (tissues without trachea), we separated them of the BmMMP1-KO mutants for knockout efficiency detections. The result showed that the knockout efficiencies of BmMMP1 were 83.3% in trachea and 86.7% in tissues without trachea (Figure 1B), which was no significant difference in both of them. It indicated that the abnormal airway of transgenic individuals was caused by BmMMP1 reduction, but not the higher knockout efficiency. We also detected the mRNA expressions of BmMMP1 in the trachea and other tissues of WT and mutants (Figure 1C). The expressions in mutants were significantly lower than in the Dazao wild type (WT). BmMMP1 had higher expression in the trachea than in the other tissues of WT, which demonstrated its importance for the trachea.

### 2.2. BmMMP1 Is Required for Tracheal Branch

**Figure 2:**
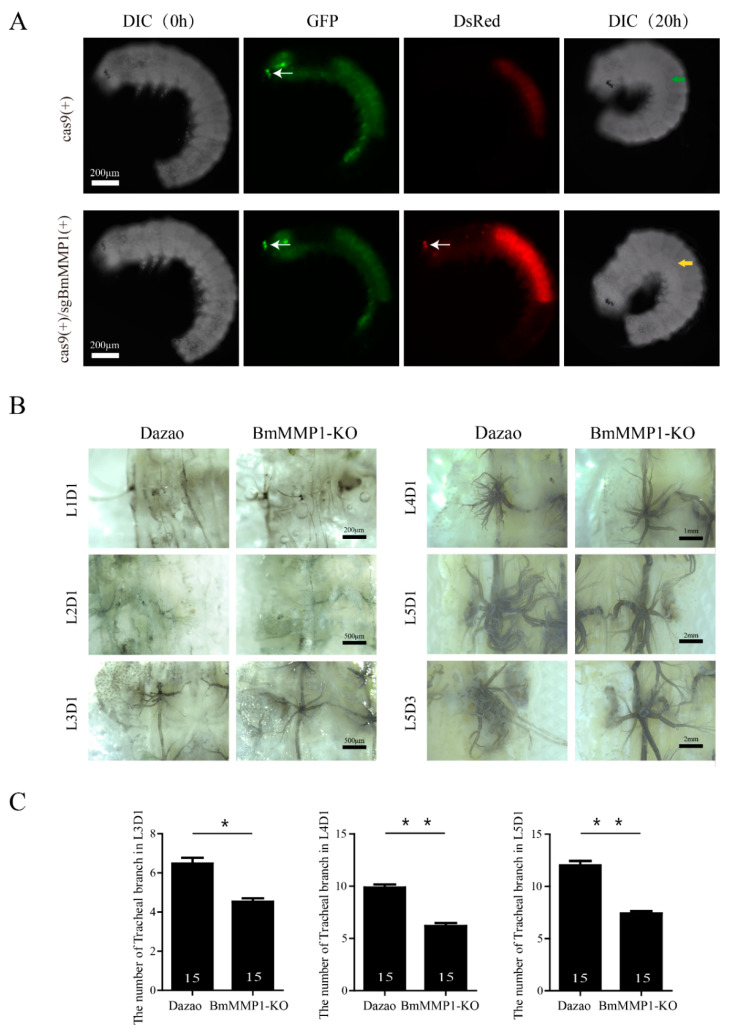
BmMMP1 is required for tracheal branching, but not morphogenesis. (**A**) The embryos of stage 24 imaged by fluorescence microscopy to identify Cas9(+)/sgBmMMP1(+) and Cas9(+) as control. DIC (0 h) represents observed at 0 h in video 1 and 2, DIC (20 h) represents observed at 20 h in video 1 and 2. White arrows indicate the fluorescence of embryo eyes. The green arrow indicated the trachea of control, and the yellow arrows indicated the trachea of mutant. (**B**) The development of trachea in the larval stages was observed under an optical microscope. L1D1–L5D3, first day of the first larval instar to the third day of the fifth larval instar. (**C**) Numbers of tracheal branches in first day of third larval instar (L3D1), first day of fourth larval instar (L4D1) and first day of fifth larval instar (L5D1). *n* = 15. (*, 0.01 ≤ *p* < 0.05; * *, *p* < 0.01).

Since tracheal morphogenesis takes place in the 25th embryonic stage from ectoderm invagination [23], we dissected silkworm eggs to obtain the 24th stage embryos in advance and observed the development of the trachea under Live Cell Imaging System for 20 h (Videos S1 and S2), and also imaged them to identify Cas9(+)/sgBmMMP1(+) and Cas9(+) as the control (Figure 2A). Interestingly, the morphologic generation appeared normal after knockout of BmMMP1, which indicated that BmMMP1 is not required for tracheal morphologic generation. Therefore, we immediately observed the development of the trachea in the larval stages under an optical microscope and found that the tracheal branching decreased with silkworm development (Figure 2B). Hence, the trachea plexus located in the second abdominal segment was used as the sample in this experiment. The numbers of tracheal branches were significantly decreased after the third larval instars (Figure 2C). Thus, these results indicated that BmMMP1 is essential for tracheal development, and its absence leads to a decrease in the number of tracheal branches in silkworm.

### 2.3. Taenidial Spacing Do Not Expand in Transgenic BmMMP1-KO Lines

**Figure 3:**
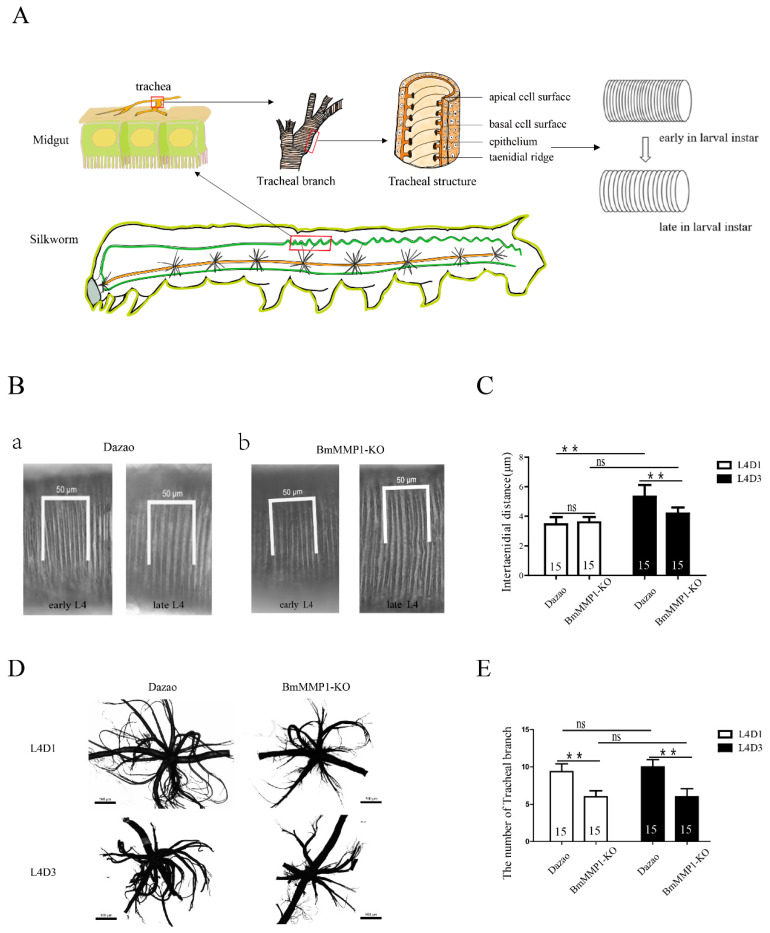
Taenidial spacing expands requires BmMMP1. (**A**) Distribution and structure of trachea of *B. mori*. The scissor images were obtained using the Easy Paint Tool SAI 1.3.6. (**B**) Tracheal cuticle expansion of WT(a) and BmMMP1-KO type(b) in the fourth larval instar. (**C**) Statistics of intertaenial distance, the white pillars represent early L4 or L4D1, the first day of fourth larval instar; the black pillars represent late L4 or L4D3, the third day of fourth larval instar. (**D**,**E**) The number of tracheal branches in early L4 and late L4. *n* = 15. (ns, *p* ≥ 0.05; * *, *p* < 0.01).

The silkworm trachea consists of a basal cell surface, epithelium, apical cell surface, and taenidium (Figure 3A), while the cuticle of the silkworm trachea, which consists of the apical cell surface and taenidium, is an important part to control tracheal extension, and the expansion of cuticle depends on the taenidial spacing expansions [24]. We observed tracheal taenidial expansion of WT (a) and BmMMP1-KO (b) in the fourth larval instar, because the trachea is bigger than that of the third instar for ease of operation and the fifth instar is not complete for BmMMP1-KO. In WT, the intertaenidial distance of the third day of the fourth larval instar (L4D3) or late L4 was wider than the first day of the fourth larval instar (L4D1) or early L4, but it was not in the BmMMP1-KO line (Figure 3B). On top of the observed differences observed, further statistical analysis was made. We measured the intertaenidial distances (a) by calculating the number (b) of taenidia in 50 (μm), using the equation, a = 50/b (μm). There was also no significant difference between early L4 and late L4 in BmMMP1-KO line, while the intertaenidial distance in late L4 is significantly wider than early L4 in WT (Figure 3C). The results shown that knockout BmMMP1 impaired the ability of the tracheal taenidium to expand.

We also counted the number of tracheal branches at the same time and found that the branch only increased during the process of tissue remodeling in interlarval molts, but there were no changes during one instar of larval growth (Figure 3D,E). The growth and development process were continued further until the trachea became thicker and larger during the molts. Combined with the expression characteristics of BmMMP1 in the trachea, the results suggest that BmMMP1 induces the increase of branches during interlarval molts and promotes the widening of taenidial spacing during the feeding period, and the latter may be the foundation of the former. It is speculated that once the taenidial expansion is blocked, the increase of tracheal branching will be affected.

### 2.4. Knockout of BmMMP1 Decreases Epidermal Cell Spacing and Breaks the Structure of Apical Membrane in Trachea

We found two main characteristics of the silkworm tracheal development: the number of tracheal branches increased during molts and taenidial expansion occurred during the larval instar. Knockout of BmMMP1 caused disruption of both processes. The taenidia belonging to the cuticle is a non-cellular structure secreted by tracheal cells, and MMPs play key roles in their development and regeneration due to their functions in the degradation of most ECM components to regulate ECM remodeling. Therefore, we examined the epidermal cell spacing and observed the structure of apical membrane.

**Figure 4:**
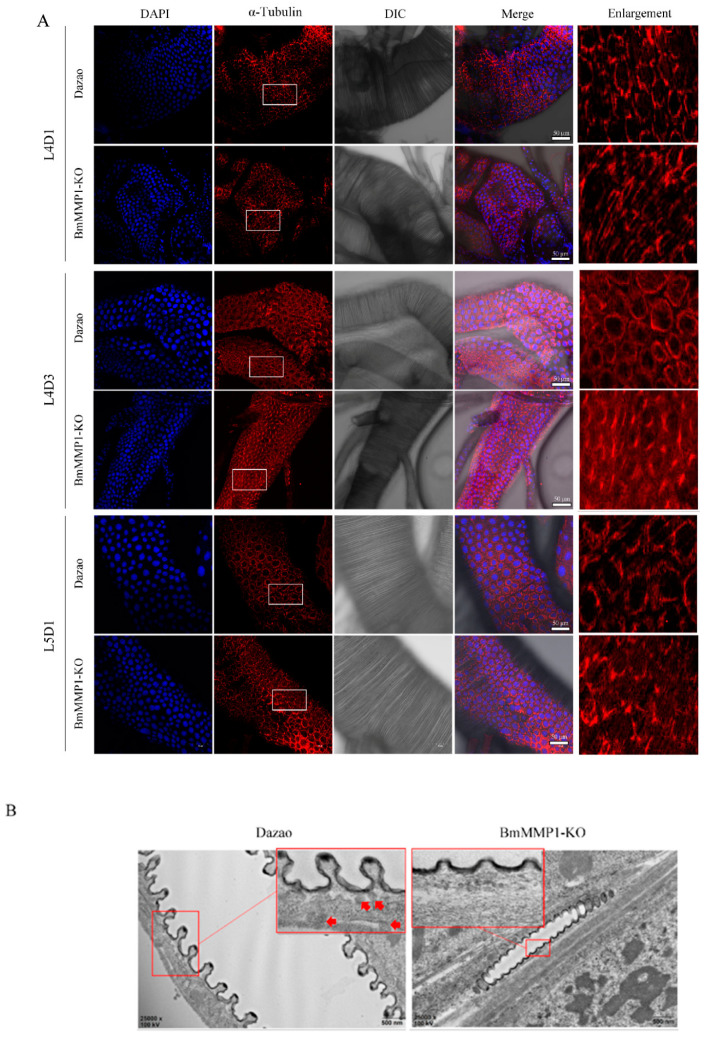
Knockout of BmMMP1 decreases epidermal cell spacing and breaks the structure of apical membrane in trachea. (**A**) Immunofluorescence assay of trachea in L4D1, L4D3, and L5D1. DAPI (Blue) stained cell nucleus, α-Tubulin (Red) stained cytoskeleton. (**B**) Remodeling of tracheal apical membrane observed by TEM. The big red box is a magnification of the small box and the red arrows indicate apical membrane protrusions.

We examined the epidermal cell spacing of the trachea in L4D1, L4D3, and L5D1 through immunofluorescence (Figure 4A), which allowed observation of the developmental changes of the trachea at one larval instar, and also allowed comparison of the developmental changes during different instars. After deletion of BmMMP1, the space between the trachea cells did not widen with tracheal development, especially in the L4D3. We visualized the apical membrane’s structure of the fifth instar trachea by transmission electron microscopy (TEM) (Figure 4B). The tracheal apical membrane displayed apical membrane protrusions in the WT, but these were absent in the BmMMP1-KO as indicated by the red arrows.

### 2.5. DE-Cadherin and Integrin β1 Are Accumulated in Trachea of Transgenic BmMMP1-KO Silkworm

**Figure 5:**
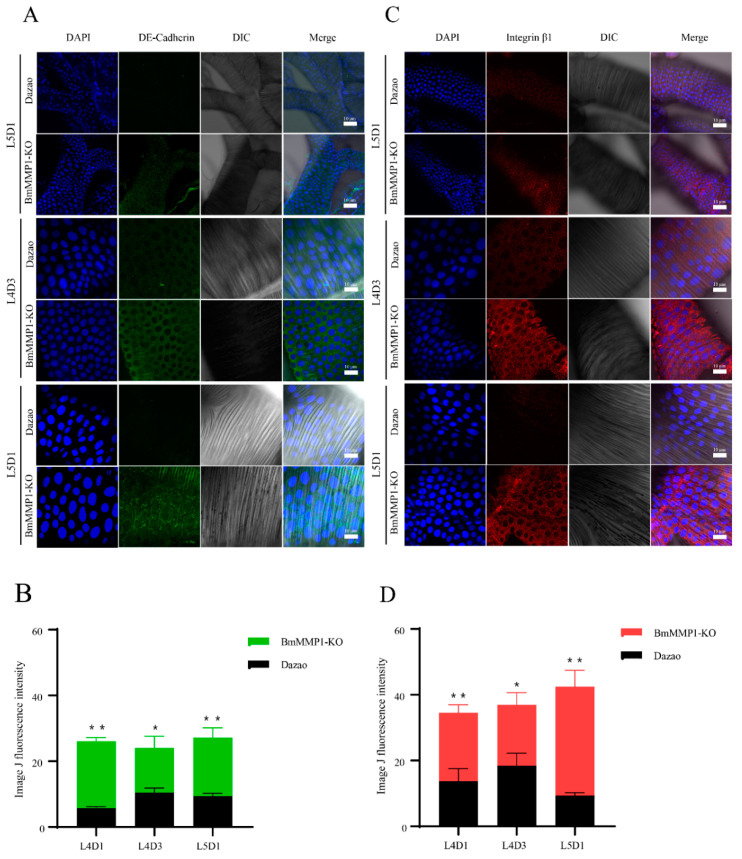
DE-cadherin and Integrin β1 accumulated in the trachea of transgenic BmMMP1-KO silkworm. (**A**) Immunohistochemistry assay for DE-cadherin of trachea in L4D1, L4D3, and L5D1. DAPI (Blue) stained cell nucleus and green fluorescence represents DE-cadherin. (**B**) Statistics of DE-cadherin fluorescence expression by ImageJ (**C**) Immunohistochemistry assay for Integrin β1 of trachea in L4D1, L4D3, and L5D1. DAPI (Blue) stained cell nucleus and red fluorescence represents Integrin β1. (**D**) Statistics of Integrin β1 fluorescence expression by ImageJ. (*, 0.01 ≤ *p* < 0.05; * *, *p* < 0.01).

Combined with the structural and developmental characteristics of silkworm trachea, we analyzed the different phenotypes of the mutants, and found that the morphology, noncellular structure and tracheal cell were all changed, and final rendering the number of tracheal branches decreased. Since the tracheal tissue has a single layer of cells and all other components were regulated by them, the growth and development of tracheal cells are particularly important for the study of BmMMP1 regulating tracheal branching [25]. We investigated the connections between tracheal cells and the basement membrane. The expressions of DE-cadherin and Integrin β1 in the silkworm trachea at the L4D1, L4D3 and L5D1 were detected by immunohistochemistry, which also allowed us to explore the development of the trachea in one larval instar and to observe the changes before and after the larval molts. The expressions of DE-cadherin and Integrin β1 were higher than WT in BmMMP1-KO (Figure 5A–C). We analyzed all fluorescence expressions by ImageJ (Figure 5B–D). The absence of BmMMP1 led to the inhibition of DE-cadherin and Integrin β1 degradation. As a result, it is speculated BmMMP1 cleaves DE-cadherin and Integrin β1 to affect tracheal branch development in the silkworm.

### 2.6. BmMMP1 Controls Proliferation and Migration of Cells in Silkworm

**Figure 6:**
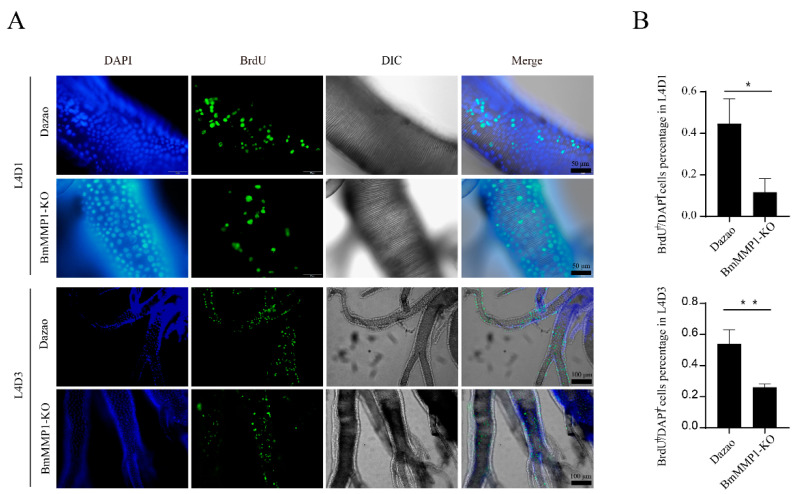
BmMMP1 affects the silkworm tracheal cells proliferation. (**A**) The proliferation activity of trachea cells by BrdU labeling assay in L4D1 and L4D3. DAPI (Blue) stained cell nucleus and green fluorescent signal BrdU positive cells or BrdU^+^ cells. Different magnification was selected for the two experiments. (**B**) The ratio statistics of BrdU^+^ cells to DAPI^+^ cells. (*, 0.01 ≤ *p* < 0.05; * *, *p* < 0.01).

**Figure 7:**
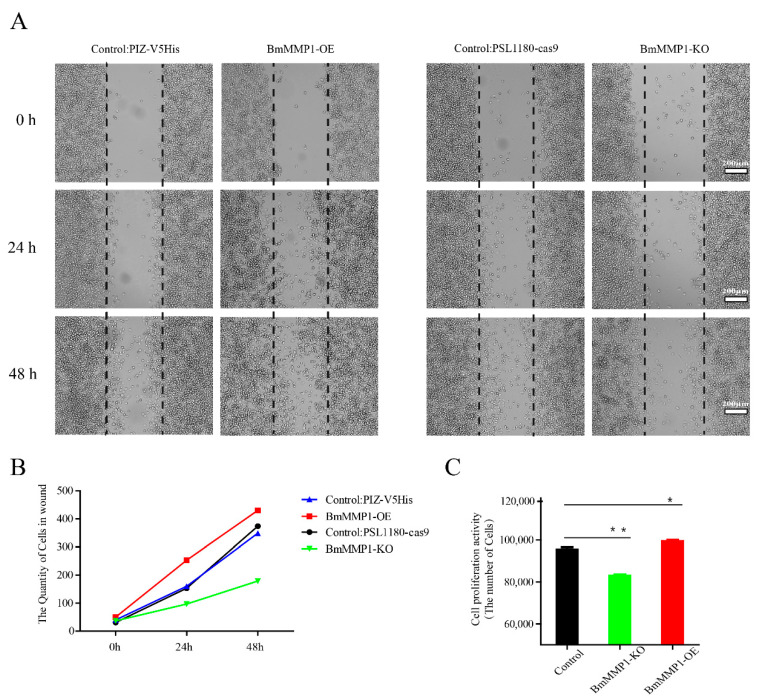
BmMMP1 affects silkworm cells migration and proliferation. (**A**) Wound healing assay in BmNS cells for 0 h to 48 h. The BmMMP1 overexpression group and knockout group have different control because of the expression vectors. (**B**) Statistics of the results of the cell wound healing assay. (**C**) MTS assay was used to determine the cell proliferation ability. Black column represented the control experimental group, green represented knockout of BmMMP1, red represented overexpression of BmMMP1. (*, 0.01 ≤ *p* < 0.05; * *, *p* < 0.01).

We tested the abilities of cell proliferation and migration in silkworm trachea and BmNS cells. We used the BrdU labeling technique to test cell proliferation of silkworm trachea in the fourth larval instar (Figure 6A). The green fluorescence signal and blue fluorescence signal were counted, respectively, and then we calculated the percentage of them. We observed that the proportion of BrdU+/DAPI+ in BmMMP1-KO was lower than WT in early fourth instar larvae and late fourth instar larvae, which indicated that knockout of BmMMP1 resulted in an obstructed proliferation of the tracheal cells. (Figure 6B). The cell wound healing assay (Figure 7A) and MTS cell proliferation assay (Figure 7B) were performed in BmNS cells, which showed that knockout of BmMMP1 reduced BmNS cell proliferation and migration abilities, while overexpression of BmMMP1 increased cell proliferation and migration abilities (Figure 7B,C). These results indicated that BmMMP1 plays an important role in regulating the proliferation and migration of silkworm cells both in vitro and in vivo. This may be a mechanism by which the matrix metalloproteinases family regulates the development of tissues.

## 3. Discussion

Tracheal tissue is necessary in multicellular organisms to exchange essential substances in and out of the body and from one part of the body to another [18,20,21,22]. In contrast to the complex trachea of mammals, the tracheal system of insects as a branching network of tubular epithelium to transport oxygen and other gases. It is an excellent model for discovering the cellular and molecular events behind the formation of the superior duct [18,26]. In our study, BmMMP1-KO mutants showed the number tracheal branches reduced, which is not the same as the phenotypes of MMPs mutations in other insects [16,27]. Therefore, studying the mechanism of BmMMP1-KO phenotypic differences in the silkworm helps explain the function of MMPs and also provides a reference for exploring the molecular mechanism behind the development of trachea and epithelial tubular tissue in insects.

In this article, we found BmMMP1 is required for silkworm tracheal branching, but not for the morphogenesis of trachea during the embryonic period (Figure 2). In *D. melanogaster*, MMPs also did not influence embryonic development but for tracheal remodeling [16]. This suggests that the function of MMP is conserved between silkworm and *D. melanogaster*. DmMMP1 and DmMMP2 regulate the development of the trachea in different ways. Knockout of DmMMP1 makes the larvae exhibited shortened dorsal trunks and DmMMP1 is required for expansion of ECM between the taenidium during each inter-molt period [16,27]. During air sac outgrowth, DmMMP2 controls branching morphogenesis through spatial restriction of FGF Signaling [17], while there are only two studies about MMPs in *B. mori*. One study reported that MMPs are involved in fat body cell dissociation and ovary development [28] and another study found that the matrix metalloproteinase genes are expressed highly during basement membrane degradation [29]. Therefore, our work provides a reference for the study of tracheal development in *B. mori*. In particular, it is a new phenotype of MMP knockout mutants that is different from the tracheal rupture in *D. melanogaster*.

In previous studies, MMPs essential roles were revealed in development and regeneration due to their functions in the degradation of most ECM components to regulate ECM remodeling [1,4]. The ECM is involved in cell adhesion, cell signaling and the structural maintenance of tissues as a complex network of proteins and proteoglycans [2,5]. During the normal events of embryogenesis and metamorphosis, tissue remodeling or cell migration through barriers must occur [30]. In this research, abnormal apical membrane remodeling was observed in BmMMP1-KO line under TEM (Figure 4). In *D. melanogaster*, MMP1 is required for expansion of ECM between the cuticle during each inter-molt period, but the mechanism is not clear [27]. DE-cadherin plays a key role in adherens junction for recruitment of other adhesion proteins to the cytoskeleton nucleation site, while the junction of cell–ECM is regulated by integrin [8,9]. DmMMP1 preferentially degrades DE-cadherin and DmMMP2 preferentially degrades basement membranes [6]. Additionally, the visceral branches migrate toward the midgut and spread over the surface of the visceral mesoderm requiring two α-integrin subunits. They both associate with the common beta-integrin subunit to assemble basal lamina for cell migration by capturing specific laminin molecules [31,32,33], while our results showed the absence of BmMMP1 leads to DE-cadherin and Integrin β1 accumulated in trachea of transgenic BmMMP1-KO silkworms. Therefore, it illustrates that BmMMP1 can cleave DE-cadherin and Integrin β1 to break cells and cell–ECM junctions to affect tracheal development in the silkworm.

In this study, knockout of BmMMP1 decreased the abilities of cell proliferation and migration, while overexpression of BmMMP1 increased them in *B. mori* (Figure 6 and Figure 7). Matrix metalloproteinase plays an important role in the invasion and proliferation of rat dermal fibroblasts and trophoblasts, and it is related to wound healing and disease occurrence [2,20,34]. Thus, it is illustrated that BmMMP1 regulates trachea development by affecting cell proliferation and maybe this is an entry point for further research on the effects of MMPs on tissue development. In mammals, MMPs are involved in the regulation of tumor-related cellular behaviors, especially their invasion and proliferation [35,36]. Therefore, using MMPs as a therapeutic target and screening drugs to inhibit MMPs has become popular in tumor therapy. Compared to the more than 20 MMPs of mammals, there are few types of MMPs in insects, and the techniques of gene interference, knockout, overexpression, and gene editing in insects are mature and easy to operate. Therefore, analyzing the mechanism of insect MMPs on its growth and development may also be helpful for tumor research.

**Figure 8:**
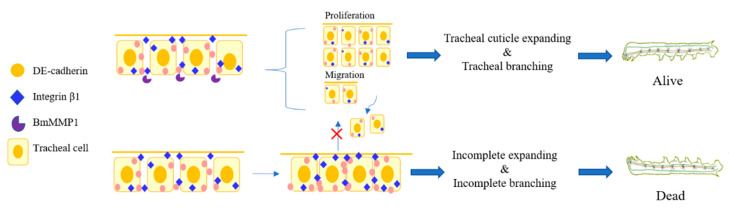
Model showing that BmMMP1 mediates tracheal branching in *B. mori*. The scissor images were obtained from Microsoft PowerPoint 2016.

In conclusion, BmMMP1 widened the cell spacing and carried out proliferation and migration through degradation of DE-cadherin and Integrin β1. This led to cell depolymerization, tracheal cuticle expansion, and ultimately promoted the increase of tracheal branches after each molt (Figure 8). The remarkable metamorphosis of insects is closely related to adapt to the complex environments. Therefore, systematic study of the influence of BmMMP1 during tracheal development has important practical significance for expanding insect resource utilization and pest control. The present researches provide a good foundation for conducting detailed studies on the molecular mechanisms involved in the regulation of silkworm trachea by BmMMP1.

## 4. Methods and Materials

### 4.1. Silkworm Strains and Cell Cultures

The *B. mori* transgenic line IE1-Cas9, BmMMP1-KO, and *B. mori* strain Dazao (control strain) were maintained at the Gene Resource Library of Domesticated Silkworm of Southwest University, Chongqing, China.

The Dazao strain *B. mori*-derived BmN-SWU1 (BmNS) cells were cultured in TC-100 insect medium (United States Biological, Swampscott, MA, USA) supplemented with 10% fetal bovine serum (Gibco, Rockville, MD, USA), penicillin (200 U/mL), and streptomycin (200 U/mL) at 27 °C.

### 4.2. Embryonic Anatomy and Live Cell Imaging System

The silkworm eggs with the embryo development up to stage 24 were dissected, and the embryos were removed in Grace insect medium (Life, Rockville, MD, USA) and cultured for observation by the Live Cell Imaging System IX83011013 (OLYMPUS, Tokyo, Japan) for 20 h. All operations were conducted under aseptic conditions.

### 4.3. Total RNA Extraction and Quantitative Real-Time PCR

Total RNA of trachea was extracted using TRIzol reagent (Invitrogen, Waltham, MA, USA) and the corresponding kit (Omega, Norcross, GA, USA). Reverse transcription was performed by using Reverse Transcriptase M-MLV (Takara, Kusatsu, Japan). Quantitative real-time PCR (qRT-PCR) was performed using the HieffTM qPCR SYBR^®^ Green Master Mix (Yeasen, Shanghai, China) and qTOWER real-time PCR system (Analytik Jena, Jena, FT, Germany). The reaction solution of qRT-PCR was 10 μL, contained 5 μL of SYBR Green, 0.8 μL of first strand cDNA template, 0.3 mM of each primer, and 5 μL ddH_2_O. The eukaryotic translation initiation factor 4A was used as the internal control, which microarray probe ID is sw22934 [37]. The primers were listed in Supplementary Table S1. Three biological replicates were performed. The secondary branch of the trachea from at least three individuals were obtained and washed three times with 1× PBS in ice bath and observed under a microscope to ensure that other tissues were cleaned.

### 4.4. Knockout Efficiency Assays

Total 500 bp of target genomic sequence that was obtained from trachea and other tissues (tissues without trachea) of the BmMMP1-KO mutants were amplified by PCR. The purified DNA was ligated into a pEASY-T5 Zero cloning vector (TransGen, Beijing, China). The positive bacteria colonies were sent for sequencing using M13 primers and counted the knockout efficiencies. All the primers were listed in Supplementary Table S1.

### 4.5. BrdU Incorporation and Immunostaining

Trachea were allowed to incorporate BrdU (Roche, Basel, BC, Switzerland) at 5 mg/mL for 3 h in Grace insect medium at 27 °C. Trachea were then fixed in 70% ethanol with 25 mM glycine for 2 h at 4 °C, and washed three times with 1× PBS. Then, the trachea were permeated with 1% Triton X-100 (Beyotime, Shanghai, China) for 30 min at 4 °C. After washed three times with 1× PBS, the trachea were blocked with 3% bovine serum albumin and 10% sheep serum in 1× PBS (blocking solution) at 4 °C for 2 h. The trachea were further incubated with anti-BrdU antibody (1:50; Roche, Basel, BC, Switzerland) in blocking solution for 5 h at 4 °C, washed three times with 1× PBS, and incubated at 4 °C for 1 h with Alexa 488-conjugated donkey anti-mouse antibody (1:500; Life, Rockville, MD, USA) in blocking solution. DNA was stained with 2, 4-diamidino-2-phenylindole (1:500; Beyotime, Shanghai, China).

The other antibodies used in this study were α-Tubulin, DE-cadherin, Integrin β1 (1:200; Beyotime, Shanghai, China) and Alexa 555 conjugated donkey antirabbit anti-body (1:500; Life, Rockville, MD, USA). The trachea were observed with a confocal microscope FV300003040108 (Olympus, Tokyo, Japan).

### 4.6. Wound Healing Assay and Cell MTS Assay

Briefly, 1 × 10^5^ BmNS cells/well in 6-well plates were allowed to reach a confluency of nearly 80% and a wound was created with a pipette tip after transfected 48 h. Cells were washed with 1× PBS to remove cell debris. Furthermore, cells were cultured in TC-100 insect medium and were observed in 0 h, 24 h, and 48 h.

The cell proliferation ability was analyzed by using a colorimetric method, MTS assay (Promega, Madison, WI, USA). Cells were collected and counted at 72 h after transfection; 5 × 10^4^ cells were placed in 96-well plates with 100 µL complete medium. A total 10 µL of MTS was added to each well and then incubated at 37 °C for 30 min. The absorbance was measured at 450 nm. All experiments were repeated at least three times.

### 4.7. Transmission Electron Microscope (TEM)

The trachea were fixed over 24 h at 4 °C in 2.5% glutaraldehyde, thoroughly washed in 0.1 M PBS, pH 7.2, post fixed in 0.5% osmium tetroxide for 2 h and embedded in resin according to manufacturer recommendations. From the fixed, embedded tissue, 70 nm sections were cut, stained in Reynold’s lead citrate, and viewed on a H7650 transmission electron microscope to observe the apical membrane (Hitachi, Tokyo, Japan).

### 4.8. Statistical Analysis

All experiments were performed at least three times. All data are expressed as the mean ± SD of three independent experiments. All of the statistically significant differences among various treatments were determined using the Student’s *t*-test. *p* ≥ 0.05 was considered no statistically significant and indicated with “ns”; 0.01 ≤ *p* < 0.05 was considered statistically significant and indicated with “*”; *p* < 0.01 was considered highly significant and indicated with “**”.

## Data Availability

Not applicable.

## Supplementary Materials

The following are available online at https://www.mdpi.com/article/10.3390/ijms22115618/s1, Figure S1: CRISPR/Cas9-mediated editing of the *B. mori* genome in sgBmMMP1×IE1-Cas9 transgenic individuals, Table S1: The primer sequences used for this study, Video S1. The tracheal morphogenesis of control took place in the 25th embryonic stage, Video S2. The tracheal morphogenesis of BmMMP1-KO mutant took place in the 25th embryonic stage.

## Abbreviations

BmMMP1-OE	BmMMP1 overexpression
BmMMP1-KO	BmMMP1 knockout
BmNS	BmN-SWU1
BrdU	5-Bromo-2′-Deoxyuridine
DAPI	2, 4-diamidino-2-phenylindole
ECM	Extracellular matrix
FGF	Fibroblast growth factor
MMPs	Matrix metalloproteinases
mRNA	Messenger RNA
MTS	3-(4,5-dimethylthiazol-2-yl)-5-(3-carboxymethoxyphenyl)-2-(4-sulfophenyl)-2H-tetrazolium
PBS	Phosphate buffer saline
qRT-PCR	Quantitative real-time reverse transcription polymerase chain reaction
TEM	transmission electron microscopy
WT	Wild type
