# Supplementary material for: A Matrix Metalloproteinase Mediates Tracheal Development in Bombyx mori
Source: Int J Mol Sci. 2021 May 25;22(11):5618. doi: 10.3390/ijms22115618 (PMC8198827; doi:10.3390/ijms22115618)
Supplement: Supplementary file 1 [file ijms-22-05618-s001.zip › Supplemental Material.pdf]

Supplemental Material

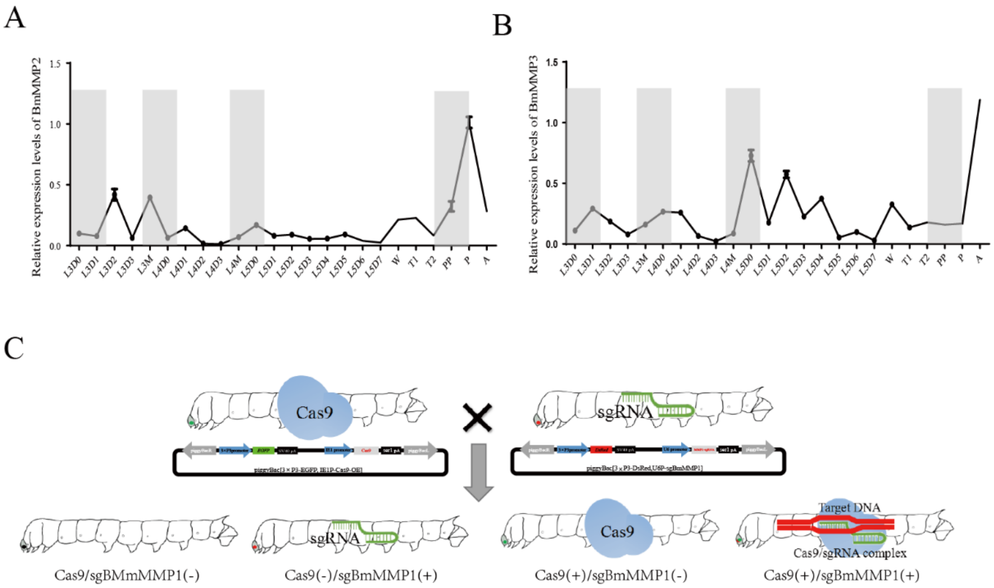

Figure S1. (**A**) The expressions of BmMMP2 mRNA levels during tracheal development in *B. mori*. (**B**) The expressions of BmMMP3 mRNA levels during tracheal development in *B. mori*. L3–L5, third to fifth larval instars; W, wandering stage; S, Spinning stage; PP, prepupal stage; P, pupal stage; A, adult stage. The gray rectangles were used to mark the critical remodeling periods of the trachea. (**C**) Schematic diagram of piggyBac [3×3P3-DsRed, U6P-sgBmMMP1] and piggyBac [3×3P3-EGFP, IE1P-Cas9] transgenic vector. Transgenic vectors were injected in silkworm eggs with the helper plasmid. The G1 generations of the IE1-Cas9 and U6-sgBmMMP1 positive lines were detected using fluorescence microscopy. The G2 generation, Cas9(-)/sgBmMMP1(-), Cas9(-)/sgBmMMP1(+), Cas9(+)/sgBmMMP1(-), and Cas9(+)/sgBmMMP1(+) transgenic hybrid lines, were generated by G1 hybridization. The Cas9(+)/sgBmMMP1(+) transgenic hybrid line is BmMMP1-KO mentioned in this study.

Supplementary Table S1. The primer sequences used for this study.

**Primers**	**Sequences** (5'-3')
QBmMMP1/F	AAGCCTTTGTCTAACTGGGATGG
QBmMMP1/R	GGGAACTGAGGGTTGTCTGTG
QBmMMP2/F	TGGACATTCGTTAGGACTTAGC
QBmMMP2/R	GTTGTTGTGGTCGTAGTGGTA
QBmMMP3/F	CTCCCGTCCAGCATCAAAGTT
QBmMMP3/R	ACCGCCAATACCTTTCCTCCT
Qsw22934/F	AACACCCCGTCCTGCTCACTG
Qsw22934/R	GGGCGAGACGTGTGATTTCCT
M13-F	GTTTTCCCAGTCACGAC
M13-R	CAGGAAACAGCTATGAC
T-BmMMP1-F	TGATGACGCTGAGATGTGGT
T-BmMMP1-R	CTTGCCGTTCTTGTAGGTAAAT
